# Structural complexity in the KCTD family of Cullin3-dependent E3 ubiquitin ligases

**DOI:** 10.1042/BCJ20170527

**Published:** 2017-11-01

**Authors:** Daniel M. Pinkas, Caroline E. Sanvitale, Joshua C. Bufton, Fiona J. Sorrell, Nicolae Solcan, Rod Chalk, James Doutch, Alex N. Bullock

**Affiliations:** 1 Structural Genomics Consortium, University of Oxford, Old Road Campus, Roosevelt Drive, Oxford OX3 7DQ, U.K.; 2 ISIS Pulsed Neutron and Muon Source, STFC, Harwell Science and Innovation Campus, Didcot OX11 0QX, U.K.

**Keywords:** BTB, crystallography, Cul3, Cullin-RING ligase, protein–protein interaction, ubiquitination

## Abstract

Members of the potassium channel tetramerization domain (KCTD) family are soluble non-channel proteins that commonly function as Cullin3 (Cul3)-dependent E3 ligases. Solution studies of the N-terminal BTB domain have suggested that some KCTD family members may tetramerize similarly to the homologous tetramerization domain (T1) of the voltage-gated potassium (Kv) channels. However, available structures of KCTD1, KCTD5 and KCTD9 have demonstrated instead pentameric assemblies. To explore other phylogenetic clades within the KCTD family, we determined the crystal structures of the BTB domains of a further five human KCTD proteins revealing a rich variety of oligomerization architectures, including monomer (SHKBP1), a novel two-fold symmetric tetramer (KCTD10 and KCTD13), open pentamer (KCTD16) and closed pentamer (KCTD17). While these diverse geometries were confirmed by small-angle X-ray scattering (SAXS), only the pentameric forms were stable upon size-exclusion chromatography. With the exception of KCTD16, all proteins bound to Cul3 and were observed to reassemble in solution as 5 : 5 heterodecamers. SAXS data and structural modelling indicate that Cul3 may stabilize closed BTB pentamers by binding across their BTB–BTB interfaces. These extra interactions likely also allow KCTD proteins to bind Cul3 without the expected 3-box motif. Overall, these studies reveal the KCTD family BTB domain to be a highly versatile scaffold compatible with a range of oligomeric assemblies and geometries. This observed interface plasticity may support functional changes in regulation of this unusual E3 ligase family.

## Introduction

The human KCTD family contains 25 soluble proteins that share a conserved potassium (*K*^+^) *C*hannel *T*etramerization *D*omain (a subtype of ‘BTB domain’) at their N-termini and have variable C-termini [1]. The core BTB fold (named after homologous regions in the proteins *B*road-complex, *T*ramtrack and *B*ric à brac) was first defined by the structure of the ‘T1 domain’ in the Shaker potassium channel AKv1.1a and comprises a three-stranded β-sheet flanked by five α-helices [2]. Structural studies of soluble BTB proteins have since shown the BTB fold to be a highly versatile domain mediating protein–protein interactions in a large number of transcriptional repressors as well as E3 ubiquitin ligases [3].

Perhaps, the most common BTB domain hetero-protein interaction is with the N-terminal domain of the cullin family protein Cul3, which recruits specific BTB-containing proteins into Cullin-RING E3 ligase (CRL3) complexes (reviewed in [4–8]). In this manner, the core BTB domain is structurally and functionally analogous to the substrate adaptors Skp1 and Elongin C, which bind to Cul1 and Cul2/5, respectively [9–11]. However, in contrast with these adaptors, the BTB family proteins are directly fused to ligand-recognition domains and therefore serve also as the substrate recognition subunits of the E3 ligase. The best characterized examples are the BTB–BACK–Kelch [10] and MATH–BTB fusions [12], whereas the KCTD family is less well understood. The CRL3 complex is completed by the RING-domain protein Rbx1, which binds to the Cul3 C-terminal domain (CTD) and recruits the E2 ubiquitin-conjugating enzyme charged with ubiquitin. Neddylation of this Cul3 domain promotes a conformational change that optimally positions the RING-E2 pair for efficient ubiquitylation of the E3-bound substrate [13].

Interest in the KCTD family of E3 ligases has increased significantly due to recent advances in the understanding of their roles in fundamental biological processes and pathologies [14]. KCTD10 is implicated in heart morphogenesis and congenital heart failure through regulation of Tbx5 [15] and Notch1 [16]. KCTD13 is a critical mediator of RhoA degradation [17] and brain development, with gene copy number variations leading to changes in brain size and associated psychiatric disorders [18]. KCTD17 regulates ciliogenesis by polyubiquitylating trichoplein [19] and its missense mutation is associated with autosomal-dominant myoclonus-dystonia [20]. Mutations in another KCTD family member SHKBP1 [(SH3-Domain Kinase Binding Protein 1)-Binding Protein 1] have been identified in cervical cancer [21] and acute myeloid leukaemia [22]. This WD40 repeat-containing protein stabilizes EGFR by disrupting the c-Cbl-CIN85 complex [23]. Interestingly, not all KCTD family proteins may function as E3 ligases [14,24]. One clade, including KCTD8, KCTD12 and KCTD16, instead, acts as auxiliary subunits of GABA_B_ receptors [25] and may contribute to mood disorders through modulating this essential neurotransmitter pathway [26].

BTB domains are widely observed forming homodimeric assemblies, which result from a common domain extension comprising an additional N-terminal β-strand and α-helix [3,27]. Their Cul3 interaction has been attributed to a 3-box motif consisting of a further C-terminal extension of two α-helices [10,12]. The KCTD family BTB domains and related T1 domains lack both extensions and can adopt distinct oligomeric structures and functions, as exemplified by the T1 domain tetramerization in the voltage-gated potassium (Kv) channels [2]. Solution studies have also suggested tetramer formation for some members of the soluble KCTD protein family including KCTD11 [28]. However, recently reported crystal structures have revealed closed pentameric assemblies for the BTB domains of KCTD5 and KCTD9 as well as both closed and open pentamers for KCTD1 [29,30]. Despite the lack of a 3-box, KCTD family proteins have also demonstrated high-affinity Cul3 interactions [24,30,31] and a 5 : 5 assembly of KCTD9/Cul3 has been recently observed by cryo-electron microscopy [30].

Here, we present novel X-ray crystal structures of the BTB domains of a further five members of the KCTD family and examine the stoichiometry and stability of their multimeric assemblies in solution. We also determine their binding to Cul3 and present structural models for their Cul3-dependent complexes based on small-angle X-ray scattering (SAXS) data. Our study reveals subtle complexity in BTB homo-oligomerization, including variable monomeric, tetrameric and pentameric crystal forms. Interestingly, Cul3 is able to induce the reassembly of a subset of these KCTD family members into 5 : 5 heterodecamers establishing a conserved architecture for these E3 ligase complexes.

## Results

### KCTD family BTB domains can adopt a wide range of oligomerization geometries

**Figure 1.**
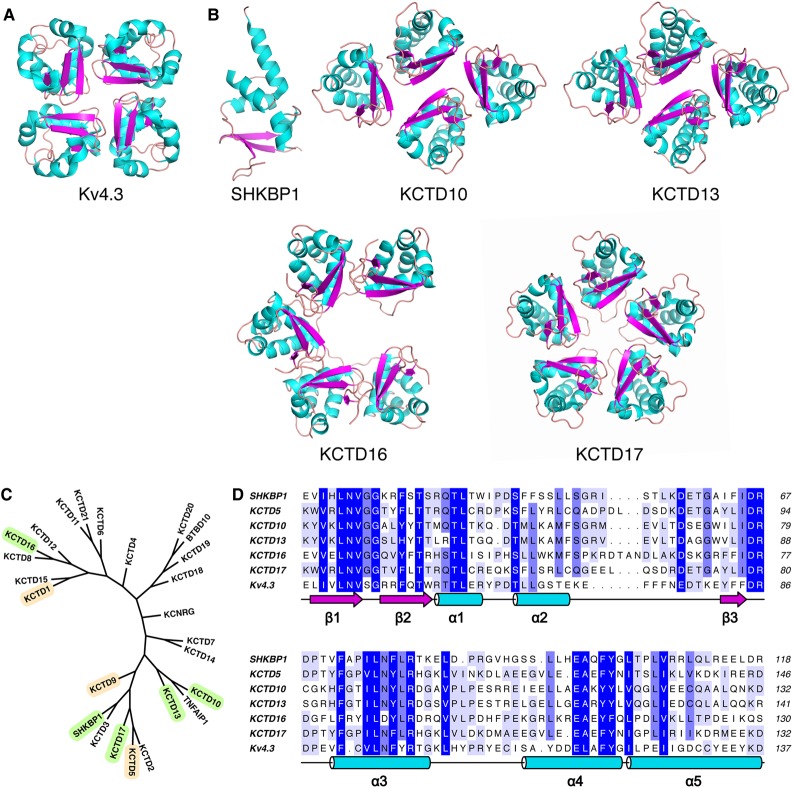
Overview of the BTB domain structures. (**A**) Previously reported X-ray structure of the four-fold rotationally symmetric tetramer of the BTB domain of human potassium channel Kv4.3 (PDB ID 1S1G). (**B**) Novel X-ray structures reported here: Monomer – SHKBP1 (4CRH); two-fold rotationally symmetric tetramers – KCTD10 (5FTA) and KCTD13 (4UIJ); C-shaped pentamer – KCTD16 (5A15); and closed pentamer – KCTD17 (5A6R). (**C**) Phylogenetic tree of the KCTD family BTB domains. The previously reported structures of KCTD1, KCTD5 and KCTD9 are highlighted in tan, and the novel structures reported here are highlighted in green. (**D**) Sequence alignment of selected BTB domains.

BTB domain structures from the KCTD family have so far been observed to form pentameric assemblies that are distinct to that of the tetrameric Kv channels (Figure 1A). To investigate the oligomerization and Cul3 binding of the remaining family members, various protein constructs encompassing the BTB domains were subcloned and tested for expression in *Escherichia coli* (Supplementary Figure S1). Five of the KCTD BTBs produced diffraction quality crystals, including SHKBP1, KCTD10, KCTD13, KCTD16 and KCTD17 (Figure 1B and Supplementary Table S1). The novel structures belong to various branches of the KCTD phylogenetic tree (Figure 1C), and have sequence similarities ranging from 30 to 77% (Figure 1D). Overall, the five BTB domain structures display a remarkable variety of oligomerization architectures. In contrast with the previous work, the expected closed pentameric structure is observed only in KCTD17, whereas the variant C-shaped pentamer, found in one crystal form of KCTD1, is also observed here in KCTD16 (Figure 1B). In contrast, a novel tetrameric assembly with two-fold rotational symmetry is observed for both KCTD10 and KCTD13 (Figure 1B). Last but not least, a further unexpected structure is observed for the BTB domain of SHKBP1, which adopts a monomeric state in the absence of its binding partner Cul3 (Figure 1B).

### Oligomerization interfaces

**Figure 2.**
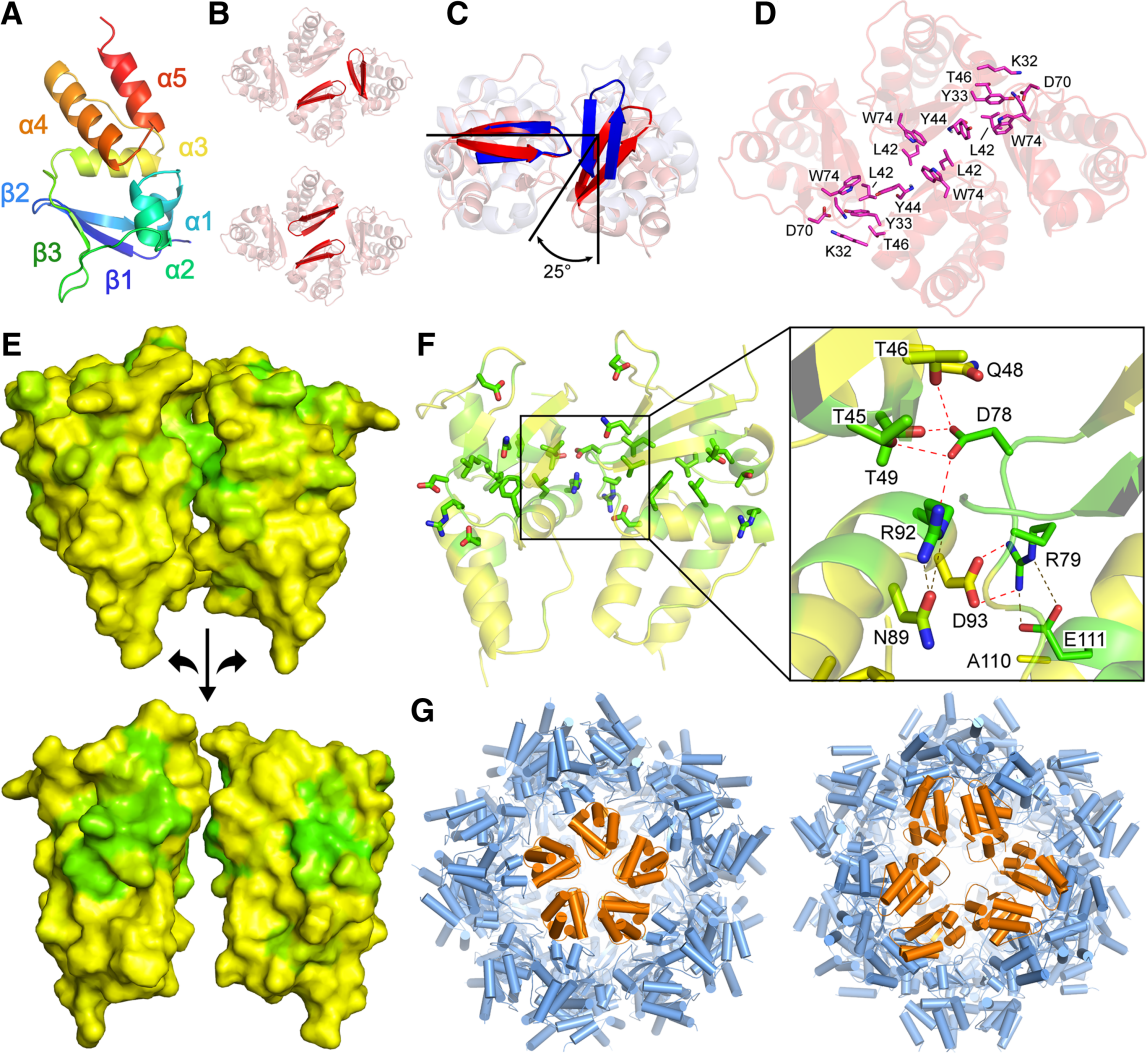
Interfaces mediating oligomer formation. (**A**) Ribbon representation of a KCTD10 monomer coloured from N-terminus (blue) to C-terminus (red). (**B**) KCTD10 tetramer with strands β1 and β2 highlighted. Top – the common interface in which the β1–β2 hairpin packs end-on to its neighbour. Bottom – in the secondary interface observed only in KCTD10 and KCTD13, the β2 strands on neighbouring molecules pack in an antiparallel fashion. The two-fold rotational axis runs directly into the page between the highlighted β hairpins. (**C**) The BTB domains of Kv4.3 (blue) and KCTD16 (red) are superimposed to indicate their different geometries at the common interface (β1–β2 hairpins are highlighted). (**D**) Ribbon representation of KCTD10 with residues at the secondary interface shown highlighted as sticks. (**E**) Surface representation of KCTD10 coloured by sequence conservation (green for most conserved and yellow for least conserved). High sequence conservation across the KCTD family is apparent at the common interface (top – as observed in the X-ray structure; bottom – BTB domains are rotated 90° with respect to the X-ray structure. (**F**) Side chains in the common interface between two KCTD10 BTB domains are shown and coloured as in panel E top. Thick and thin sticks highlight the residues from the BTBs on the left and right, respectively. Hydrogen bonds are shown as dashed lines with bonds across the interface shown in red and within a BTB in grey. (**G**) The icosahedral KCTD17 60-mer is shown as a cartoon with cylindrical helices. On the left, the pentamer mediated by the common interface is highlighted in orange. On the right, BTBs involved in the secondary interface are highlighted in orange.

The BTB fold of the Kv and KCTD families has a roughly pyramidal wedge shape and consists of five α-helices and a single β-sheet made from three β-strands (Figure 2A). With the exception of monomeric SHKBP1, each protein oligomerizes through a common interface which can be easily visualized through the characteristic β1–β2 hairpin of one monomer packing end-on to that of a neighbouring molecule (Figure 2B). The angles between monomers in their assemblies vary from 90° for Kv4.3 to ∼65° for KCTD16 (Figure 2C), giving rise to observed oligomerization geometries that range from compact to splayed. Each of these interfaces contributes ∼600 Å^2^ of buried surface area, or ∼10% of the total monomer surface area. The region of highest conservation in the KCTD family includes both sides of this interface, as well the hydrophobic core between them (Figure 2D,2E). As first described for KCTD5, the two interacting surfaces include conserved charge–charge and hydrogen bond interactions (Figure 2F).

A second interface is additionally evident in the tetrameric structures of KCTD10 and KCTD13 as visualized by an antiparallel arrangement of two β1–β2 hairpins (Figure 2B,2D). However, there does not appear to be direct interchain β-sheet hydrogen bonding. Whereas the canonical interface contributes 1210 Å^2^ to the total tetramer interface in KCTD10, the second interface contributes 940 Å^2^. We also observed higher-order packing in the crystal lattice of the His6-KCTD17 structure resulting in dodecamerization of the core pentameric ring (Figure 2G). This assembly establishes an extraordinary T = 1 icosahedral protein shell comprising 60 monomers that enclose a large water-filled cavity.

### BTB domain assembly in solution

**Figure 3.**
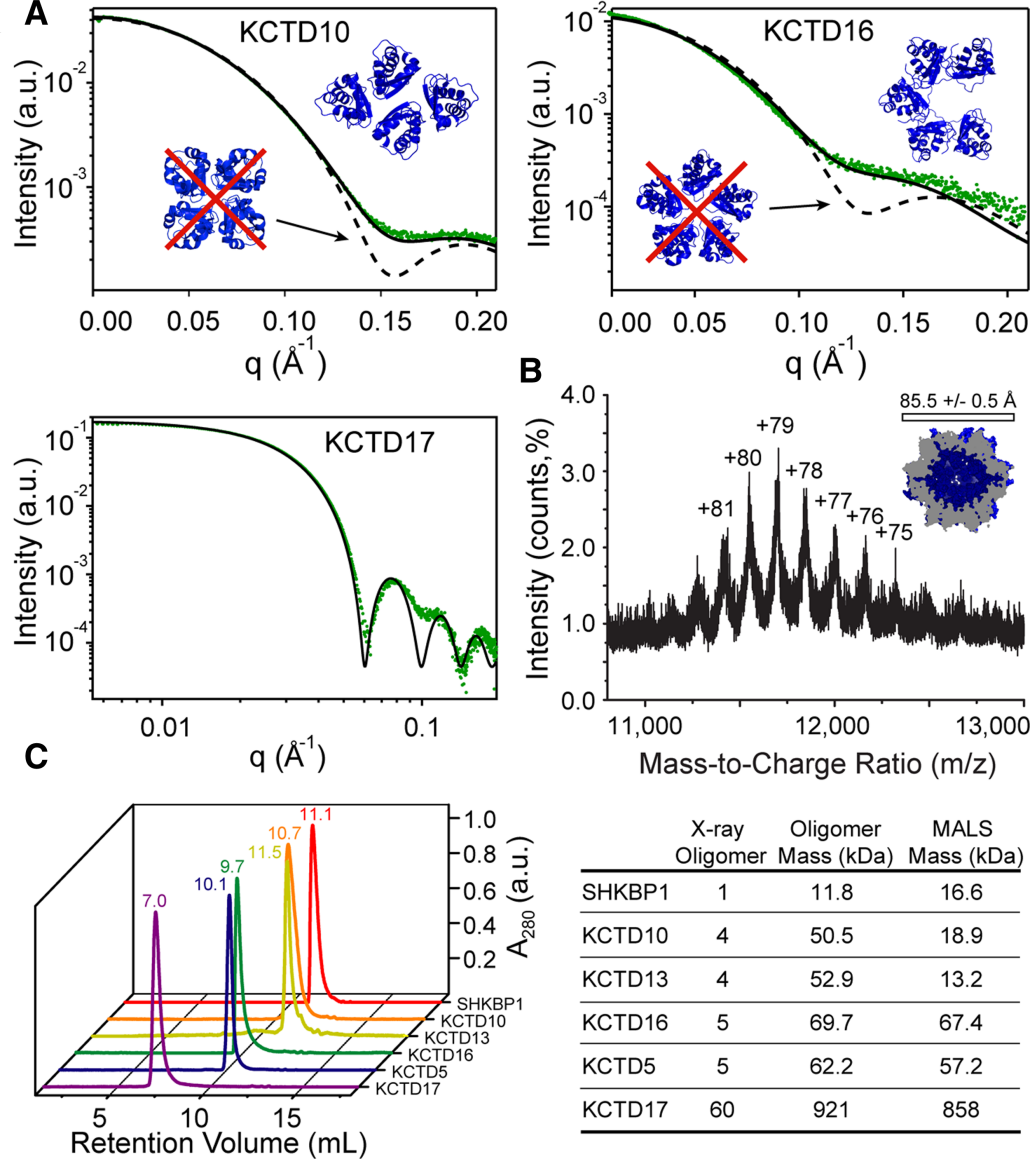
Solution states of BTB domains. (**A**) Small angle X-ray scattering of indicated BTB domain assemblies. Data are shown in green and scattering calculated from the particles observed in the X-ray structures shown as black lines. Upper left – scattering calculated for the KCTD10 tetramer (5FTA, solid black line) is compared with scattering calculated for the distinct structure of Kv 4.3 (1S1G, dashed black line). Chi value for SAXS fit – 2.2 asymmetric tetramer, 0.8 symmetric tetramer. Upper right – scattering calculated for the KCTD16 C-shaped pentamer (5A15, solid black line) is compared with scattering calculated for the closed KCTD5 pentamer (3DRZ, dashed black line). Chi value for SAXS fit – 1.8 open pentamer, 0.8-closed pentamer. Lower left – scattering calculated for the icosahedral structure of 6His-KCTD17 is shown (5A6R, solid black line). Reduced Chi square for SAXS fit – 0.53. A red cross denotes that this assembly was not observed. (**B**) Native mass spectrum of 6His-KCTD17. Charge states corresponding to KCTD17 60-mer ions (neutral mass 920 856 Da) are indicated. A charge radius value of 85.5 Å was calculated from independent MS analyses using +79 as the most abundant ion and the method of Testa et al. [32]. The calculated value of 85.5 Å was in excellent agreement with the observed radius in the crystal structure of 84 Å. The expected mass of the complex is the observed monomer mass (15347.7 Da) multiplied by 60 = 920 862 Da. The observed mass of the complex is *m/z* 11699.4 × 79 − 79 = 924173.6 Da. This corresponds to an observed mass difference of 0.35%. (**C**) Results of SEC–MALS. Left – UV (280 nm) traces of BTB domains. Retention volumes corresponding to peaks are indicated. Right – masses derived from multi-angle laser light scattering measurements are compared with masses calculated from X-ray structures.

To determine whether the oligomerization architectures observed in the crystal structures are also found in solution, a combination of size-exclusion chromatography (SEC) combined with multi-angle light scattering (SEC–MALS), native mass spectrometry (native MS) and SAXS techniques were employed. Although SHKBP1 was prone to aggregation at the concentrations needed for SAXS (data not shown), the solution X-ray scattering curves for the other BTBs were found to be monodisperse by the Guinier method (Supplementary Figure S2) and to be consistent with those calculated from the particles observed in their respective X-ray crystal structures (Supplementary Table S2). For instance, the scattering curve for KCTD10 was virtually identical with that calculated for the two-fold symmetric tetramer observed in its crystal, while being distinct from the predicted solution scattering curve for a symmetric tetramer such as that observed for the T1 domain found attached to potassium channels (Figure 3A). Modest deviations at high q for KCTD16 may be attributed to flexibility and solvation effects not accounted for in the calculated scattering. For 6His-KCTD17, the stability of the 60-mer was confirmed both in solution by its distinctive hollow sphere SAXS pattern (Figure 3A), as well as by its electrospray native MS signature (Figure 3B). Indeed, the MS analysis method of Testa et al. [32] indicated a charge radius of 85.5 Å in excellent agreement with the observed radius in the crystal structure of 84 Å.

To investigate the oligomerization further, each BTB domain was analysed using SEC–MALS (Figure 3C). The control protein KCTD5 and KCTD16 were observed to migrate as the expected pentamers, whereas 6His-KCTD17 was observed as a large species consistent with the 60-mer. In contrast, SHKBP1 was observed to migrate as a mixture of monomers and dimers, indicating only a weak propensity for multimerization. Interestingly, KCTD10 and KCTD13 also exhibited retention times and MALS scattering that were consistent with mixtures of monomers and dimers, indicating that these crystallographic tetramers were not stable in solution at lower protein concentrations (<50 µM monomer concentration compared with ∼750 µM used in SAXS experiments).

**Figure 4.**
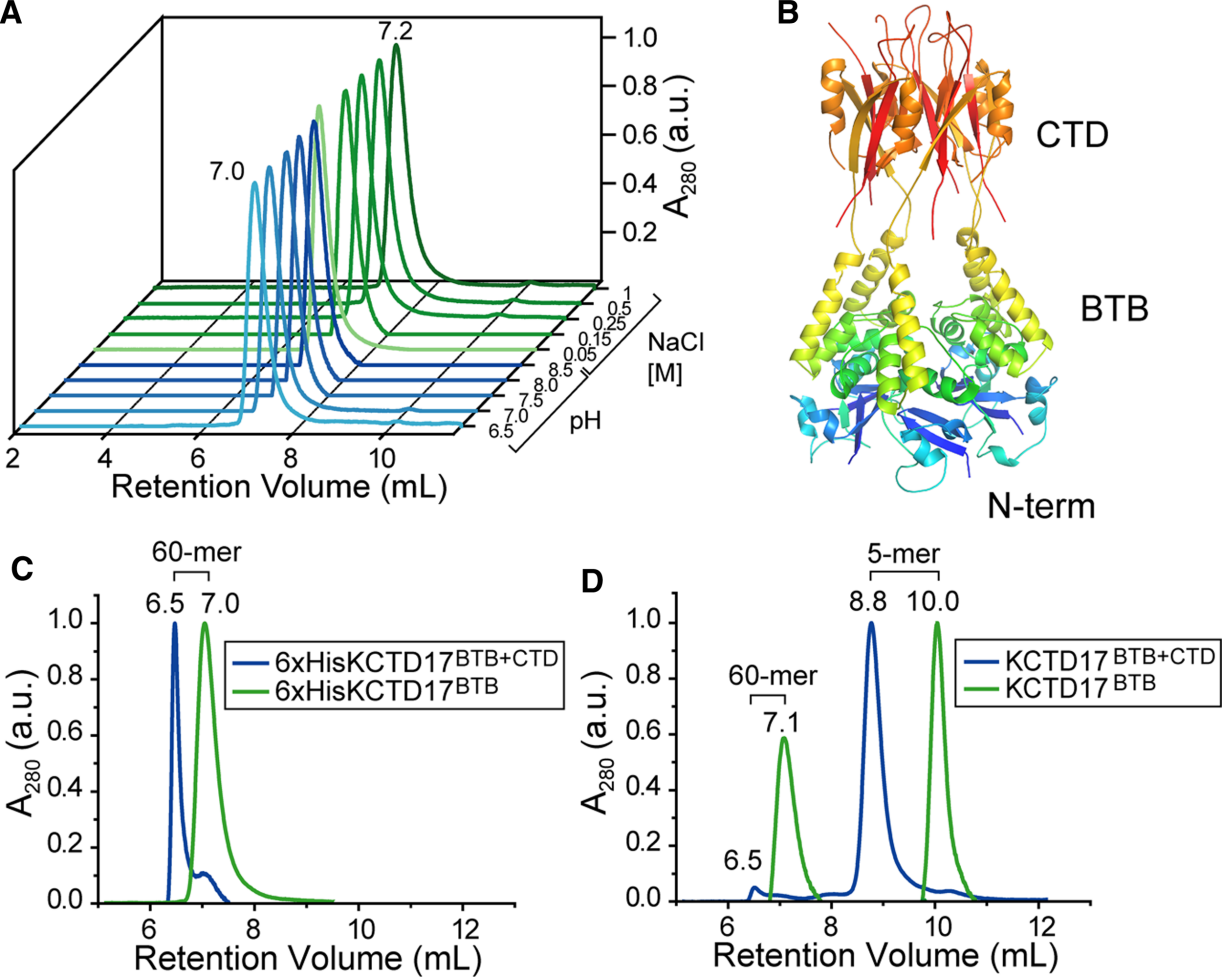
Determinants of KCTD17 60-mer stability. (**A**) Size-exclusion traces (UV 280 nm) of 6xHis-KCTD17^BTB^ under various running buffer conditions (full buffer conditions described in methods). (**B**) Homology model of multi-domain KCTD17 based on the known structure of KCTD5 [29]. (**C**) Size-exclusion traces (UV 280 nm) of indicated 6xHis-tagged KCTD17 constructs. (**D**) Size-exclusion traces (UV 280 nm) of indicated tag-cleaved KCTD17 constructs.

Finally, given the surprising assembly of our 6His-KCTD17 construct as a stable solvent-filled protein shell, we investigated the effect of pH and salt concentration, as well as construct details, on shell stability. Our crystallization construct comprising the 6His-BTB domain proved to be remarkably stable with respect to both pH and salt concentration (Figure 4A). A multi-domain construct, including the predicted C-terminal domain (KCTD17^BTB+CTD^, Figure 4B), was similarly observed as a stable 60-mer (Figure 4C). However, the shell was not stable upon the removal of the N-terminal 6His tag for the longer construct (Figure 4D), indicating that the 60-mer shell is unlikely to be physiological.

### KCTD family BTB domains exhibit a range of Cul3-binding affinities

**Figure 5.**
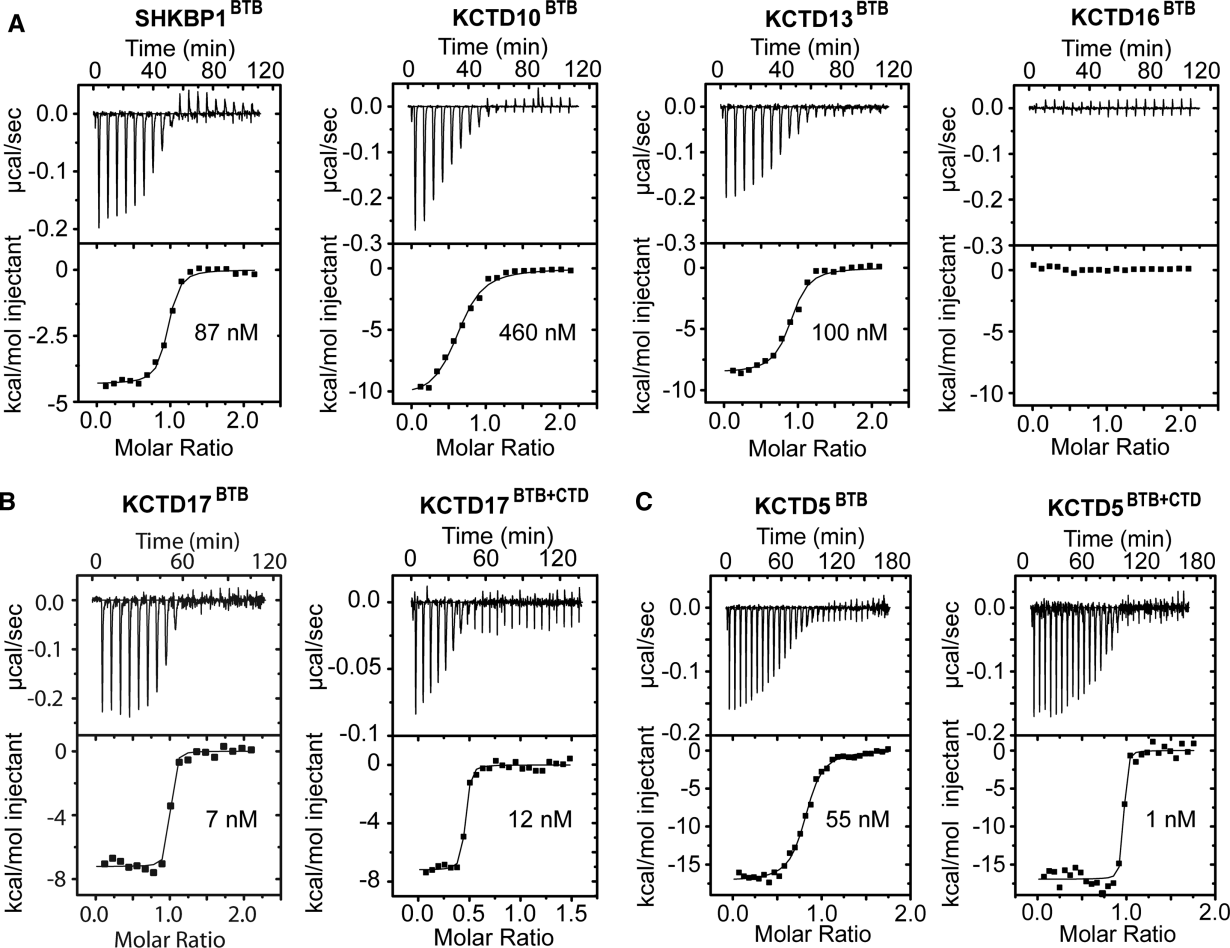
ITC measurements of Cul3 binding to KCTD family proteins. (**A**) Data for selected isolated BTB domains. (**B**) Comparison of single and multi-domain constructs of KCTD17. (**C**) Comparison of single- and multi-domain constructs of KCTD5.

BTB domain interaction with Cul3 is required for certain KCTD family members to assemble into CRL3s. To investigate the potential for such interaction, the binding of the KCTD proteins to the Cul3 N-terminal domain was assessed by isothermal titration calorimetry (ITC). Binding was observed for each of the BTB domains with the exception of KCTD16, which showed no evidence of Cul3 interaction (Figure 5A). Overall, the BTB domains displayed a range of affinities from low-to-high nanomolar: KCTD17 (*K*_d_ = 7 nM) > KCTD5 (*K*_d_ = 55 nM) > SHKBP1 (*K*_d_ = 87 nM) > KCTD13 (*K*_d_ = 100 nM), KCTD10 (*K*_d_ = 460 nM) (Figure 5A–C and Supplementary Table S3).

We additionally assessed the binding of longer constructs of KCTD5 and KCTD17 that could be recombinantly expressed with their intact CTD region. For KCTD17, the binding affinity was effectively unchanged, although this approached the limit of measurement (Figure 5B). However, for KCTD5 there was an apparent 50-fold improvement in binding (*K*_d_ = 1 nM; Figure 5C), indicating that the CTD enhances the BTB–Cul3 interaction, perhaps through strain-induced organization of Cul3 binding sites at the BTB–BTB interfaces.

### KCTD family proteins bind Cul3 as 5 : 5 heterodecamers

**Figure 6.**
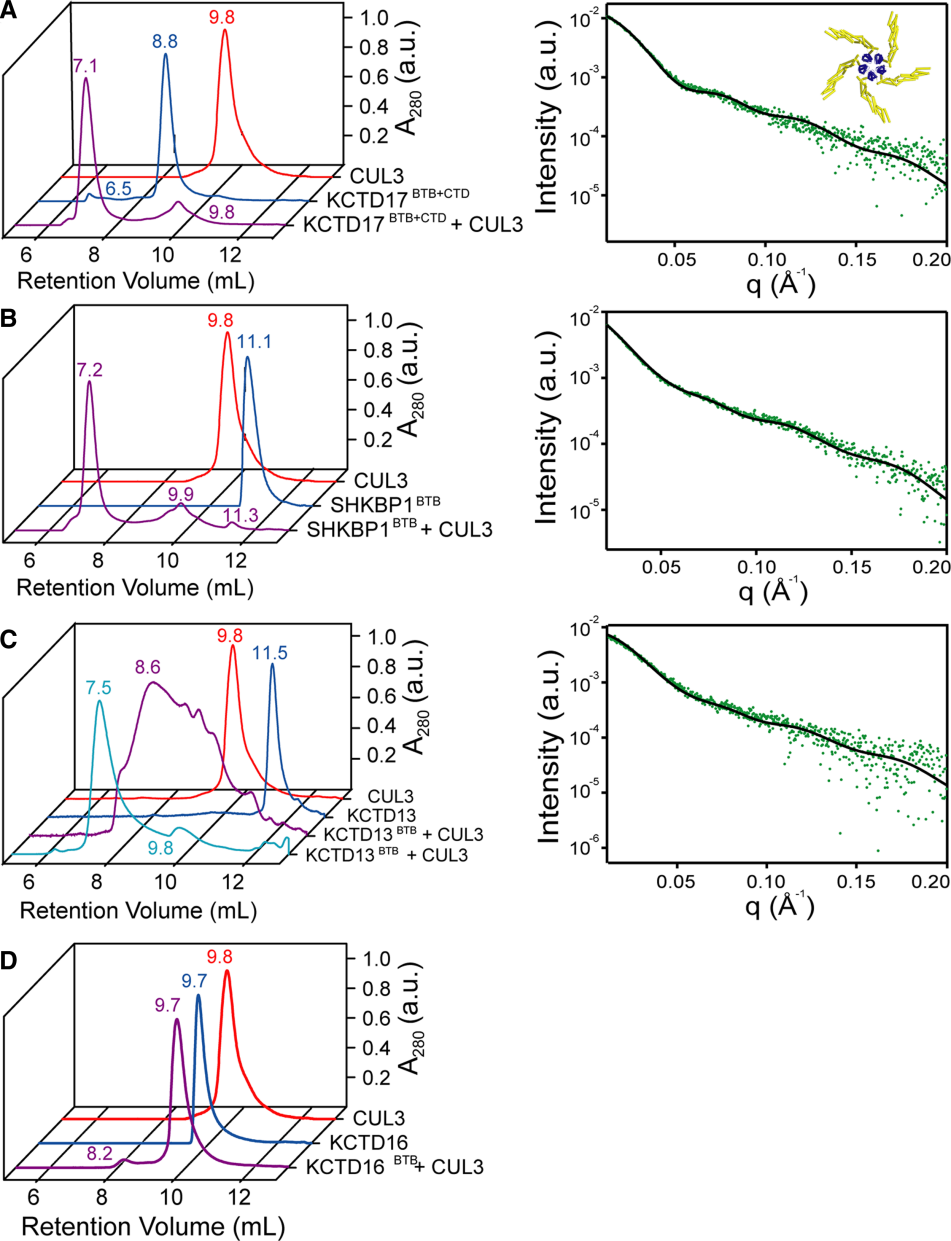
KCTD family BTB domains bind to Cul3 as 5 : 5 heterodecamers. Left panels – size-exclusion chromatograms (UV 280 nm) of indicated KCTD proteins alone and in complexes with Cul3. Right – small angle X-ray scattering of indicated KCTD proteins in complex with Cul3. Data are shown in green and scattering calculated from the inset model shown as a solid black line (BTBs - blue cartoon; Cul3 – yellow cartoon). (**A**) Data collected using the multi-domain KCTD17^BTB+CTD^ construct. Chi value for SAXS fit – 2.9 (**B**) Data collected using the BTB domain of SHKBP1. Chi value for SAXS fit – 2.2. (**C**) Data collected using the BTB domain of KCTD13. KCTD13 was prone to aggregation (as indicated by the purple line in the size-exclusion chromatography). A further size-exclusion chromatography step was needed for homogeneity (light blue line). Chi value for SAXS fit – 2.1. (**D**) Size-exclusion chromatograms (UV 280 nm) of KCTD16 alone and in the presence of Cul3 showing no apparent binding.

**Figure 7.**
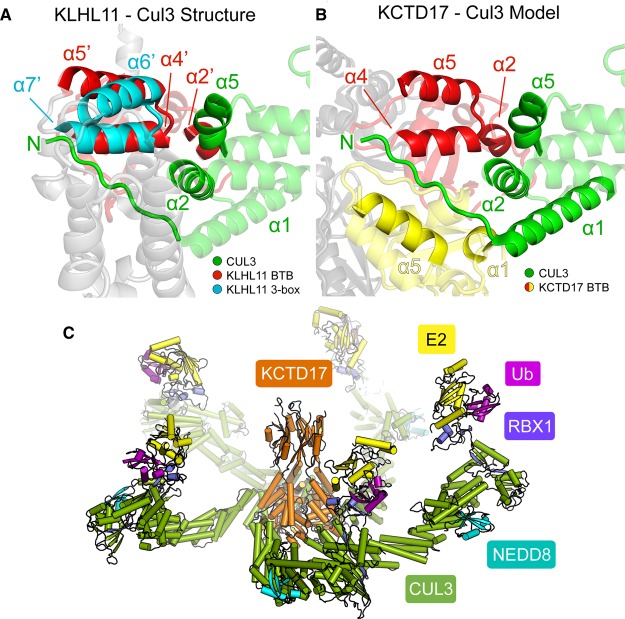
Model of Cul3 binding to KCTD proteins. (**A**) View of the known KLHL11-Cul3 structure highlighting the interface positions of the KLHL11 BTB (red) and 3-box (light blue) domains (PDB ID 4AP2) [10]. Other C-terminal KLHL11 regions are shown in grey while Cul3 is shown in green. (**B**) Homology model of the KCTD17–Cul3 interface based on the conserved BTB domain regions of KCTD17 and KLHL11. The Cul3 subunit (coloured green) binds to the equivalent BTB domain of KCTD17 (red), but also forms additional contacts with an adjacent BTB domain (yellow) in the KCTD17 pentameric ring. (**C**) Model of the KCTD17 ubiquitin ligase complex with charged E2-ubiquitin pairs. Remaining parts of the complex were modelled from other homologous structures (PDB IDs 3DQV [13], 1LDK [11], 4AP4 [47]). An alternative view from the top of the complex is presented in Supplementary Figure S3.

Efforts to co-crystallize the KCTDs in complex with Cul3 for structure determination were unsuccessful. Their binding mode was therefore investigated further by SEC as well as by SAXS to provide insight into the size and shape of these complexes, respectively. To investigate KCTD17, we utilized a tag-cleaved form of the larger BTB–CTD multi-domain construct to ensure a homogeneous sample free from any KCTD17 60-mers. As expected, the complex of KCTD17 and Cul3 was found to run on SEC with a radius of hydration consistent with the formation of a 5 : 5 KCTD to Cul3 heterodecameric assembly (Figure 6A). Analysis of the SAXS data revealed that the scattering curve fit well to that of a structural model built by superposition of each KCTD17 BTB subunit with a homologous BTB domain from the structure of the KLHL11-Cul3 complex [10] (Figures 6A and 7A). This model placed the Cul3 subunits at the interface between two BTB subunits increasing the overall interaction surface (Figure 7B).

We additionally used SEC and SAXS to investigate the more atypical SHKBP1, KCTD13 and KCTD16 BTB domains, which had previously demonstrated non-pentameric structures when studied in isolation. We observed that the monomeric SHKBP1 BTB domain was also stably incorporated into a 5 : 5 heterodecamer when mixed with Cul3 protein. Moreover, the SAXS data from this complex displayed an identical scattering curve to the KCTD17-Cul3 complex (Figure 6B). A rather different architecture might be expected for the BTB domain of KCTD13, which crystallized as a two-fold symmetric tetramer (Figure1B). This protein was prone to aggregation and produced a heterogeneous sample upon mixing with Cul3 (Figure 6C). However, a more homogeneous KCTD13-Cul3 complex could be prepared by taking the early-eluting fractions of the complex mixture and re-injecting them onto a size-exclusion column to isolate the largest species. SAXS experiments using this sample produced a scattering curve fitting the same 5 : 5 heterodecamer structural model as the KCTD17-Cul3 and SHKBP1-Cul3 complexes (Figure 6C). In contrast, KCTD16 again demonstrated no interaction with Cul3 (Figure 6D). Overall, these data suggest that Cul3 can promote a common pentameric packing arrangement in the KCTD family by binding across the BTB–BTB interfaces.

## Discussion

In this report, we present X-ray crystal structures of five novel BTB domains from the KCTD family that reveal previously unobserved oligomeric forms and geometries. Importantly, four of the structures explore new phylogenetic clades within the KCTD family (Figure 1C). As defined by Skoblov et al. [14], these include clade C (KCTD10 and KCTD13), clade D (SHKBP1) and clade F (KCTD16), while the remaining protein KCTD17 belongs in clade E with the previously characterized protein KCTD5. Interestingly, the multimerization behaviour of the crystallized proteins separates well by evolutionary clade. For instance, the closed pentameric assembly previously observed for KCTD5 [29] was also found in the KCTD17 structure. Both KCTD5 and KCTD17 have demonstrated E3 ligase activity; KCTD5 down-regulates GPCR signalling by targeting Gβγ heterodimers for degradation [33], whereas KCTD17 controls ciliogenesis by inducing degradation of trichoplein [19].

The BTB domain of SHKBP1 is the next closest paralogue in our study and is located in the phylogenetic tree between KCTD5 and KCTD9 (Figure 1C), the latter of which has also been crystallized as a closed pentamer [30]. The SHKBP1 BTB domain adopted a surprising monomeric structure in isolation, but could form the expected 5 : 5 heterodecamer when mixed with Cul3. Unusually, the small CTD common to most KCTD family members is replaced in SHKBP1 by a large WD40 repeat domain, a substrate-binding domain also present in many F-box family E3 ligases, such as β-TrCP1 [34]. It will be interesting in future to determine how these distinct families with different oligomeric states compare in their mechanisms of substrate capture and ubiquitylation.

Evolutionarily distant from these family members are KCTD16 and KCTD1, which belong to closely related clades F and A, respectively [14]. Neither protein binds to Cul3 and both have now been observed to form unusual C-shaped pentamers by X-ray crystallography [30]. We show here that this geometry is also maintained for the KCTD16 BTB in solution. Nevertheless, a second crystal form of KCTD1 has demonstrated that it can additionally form a closed pentameric structure [30]. The clade F proteins KCTD16, 12 and 8 bind to the cytoplasmic tails of the GABA_B2_ GPCR, acting as auxiliary subunits that modulate membrane polarization dynamics of cells in response to GABA stimulation [25]. Plasticity within the BTB interfaces of some KCTD family groups might therefore reflect an important adaptation for their biological function.

Our crystal structures of KCTD10 and KCTD13 represent the first structures of family members from clade C. Unexpectedly, both proteins crystallized as two-fold symmetric tetramers revealing a different complex architecture to other KCTD families characterized to date. These structures were also notably distinct from the four-fold rotationally symmetric tetramer of the Kv4.3 BTB domain. However, KCTD10 and KCTD13 were both dissociated in solution at lower protein concentrations, demonstrating that their BTB domain interfaces were weak, as also apparent for SHKBP1. This likely explains how Cul3 addition can drive the reassembly of these proteins into 5 : 5 heterodecamers. Consistent with this assembly, KCTD13, also known as BACURD1, has been identified as an E3 ubiquitin ligase mediating the ubiquitination and proteasome-dependent destruction of RhoA [17].

The structural basis for Cul3 binding to KCTD proteins remains to be fully elucidated at high resolution. General insights into the BTB–Cul3 interaction have been obtained from recent crystal structures of Cul3 in complex with other BTB-containing E3 ligases, including SPOP [9], KLHL11 [10] and KLHL3 [35]. These proteins exemplifying the MATH-BTB and BTB-BACK-Kelch family E3 ligases have an additional important Cul3 recognition element just C-terminal to the BTB domain, coined the ‘3-box', that binds to an N-terminal extension in Cul3 and increases the binding affinity by up to 30-fold [10]. The 3-box is absent from the KCTD family proteins suggesting that they may adopt additional alternative interactions. Homology modelling based on the conservation between KCTD17 and the KLHL11-Cul3 structure suggests that the close proximity of a neighbouring BTB domain in the KCTD17 pentamer can provide an additional Cul3-binding interface that may substitute for the lack of a 3-box (Figure 7). This model is supported by cryo-EM studies [30] and likely explains how Cul3 is able to ‘glue together’ SHKBP1 monomers into a 5 : 5 heterodecamer by forming contacts across the BTB interfaces. However, induced conformational changes in the BTB domains upon Cul3 binding cannot be strictly ruled out.

In addition to the BTB domain, KCTD family proteins contain a CTD, which in KCTD5 is observed to pentamerize into a beta-propeller geometry. The CTDs are varied between clades. For instance, the CTDs of KCTD5, KCTD2 and KCTD17 (clade E) are predicted to be different from those of KCTD10 and KCTD13 (clade C) [29]. While BTB domain structures have shown variable assemblies, oligomerization of the associated CTDs will also exert an additional influence on the overall oligomerization properties of the full-length proteins. To date, the only reported detailed structural characterization for a multi-domain construct has been for KCTD5, which displays an axially symmetric pentameric structure [29]. It remains unclear if all KCTD family members would form KCTD5-like pentameric assemblies, or if for instance full-length KCTD10 or KCTD13, which belong to a relatively diverged clade, would retain tetrameric forms similar to their isolated BTB domain structures. Unfortunately, we were unable to recover protein from these multi-domain constructs from either bacterial or baculoviral recombinant expression systems. Nonetheless, further studies to investigate the native assemblies of the KCTD family proteins are warranted. In particular, whereas the BTB domains mediate KCTD multimerization and Cul3 binding, it is the CTDs that impart substrate specificity to these E3 ligases.

Additionally, our serendipitous discovery that a construct of 6His-KCTD17 forms a robust icosahedral 60-mer may be of interest for protein design applications, such as a vehicle for multivalent peptide display or for carrying cargo internally. The C-termini of the monomers are pointed outwards from the external surface of the spherical body, making the construct amenable for multivalent display by C-terminal fusion of desired sequences. Moreover, the small 13 kD size of the construct and its abundant expression in *E. coli* make it a good candidate for such purposes.

Overall, the pyramidal wedge shape of the KCTD family BTB domain creates a versatile scaffold compatible with a range of oligomeric assemblies and geometries that may impact function. The present study shows that these properties diverge unexpectedly across the KCTD family, but cluster into different phylogenetic clades. Finally, for the subset of E3 ligases, a recurrent structural model is emerging in which a central substrate-binding site is surrounded by an outer ring of charged E2-ubiquitin conjugates seemingly poised for efficient ubiquitination of diverse substrate sizes and geometries (Figure 7C). Identification of these substrates and their binding modes forms the next challenge in our understanding of this unusual protein family which has important links to cancer and neurological diseases.

## Experimental procedures

### DNA constructs

The cDNAs for human SHKBP1 (UniProt Q8TBC3, ‘BTB' – residues G18-S120), KCTD5 (UniProt Q9NXV2, ‘BTB' – residues G40-R145/‘BTB + CTD' - residues G40-M234), KCTD10 (UniProt Q9H3F6, ‘BTB' – residues G26-E135), KCTD13 (UniProt Q8WZ19, ‘BTB' – residues G27-L144), KCTD16 (UniProt Q68DU8, ‘BTB' – residues G16-E133), KCTD17 (UniProt Q8N5Z5, ‘BTB' – residues G20-K131/‘BTB + CTD' – residues G20-Q220) were cloned using ligation-independent cloning into the vector pNIC28-Bsa4 for expression as N-terminally hexahistidine-tagged (MHHHHHHSSGVDLGTENLYFQSM) proteins [36]. DNA sequencing of the constructs revealed a F61L mutation in KCTD10 affecting the hydrophobic core. The human Cul3 construct in the vector pNIC-CTHF has been described previously (UniProt Q13618, ‘NTD' – residues M1-L388) and contains the engineered mutations I342R and L346D introduced to stabilize the isolated Cullin-repeat domain [10].

### Protein expression and purification

Expression was carried out in the *E. coli* strain BL21(DE3)R3-pRARE2. Cultures were grown in LB medium supplemented with 100 µg/ml ampicillin to an OD_600_ of 0.6 at 37°C, and protein expression was carried out overnight at 18°C by induction with 0.4 mM isopropyl 1-thio-β-d-galactopyranoside. Cells were harvested by centrifugation, resuspended in a binding buffer (50 mM HEPES pH 7.5, 500 mM NaCl, 5% glycerol, 5 mM imidazole) and lysed by sonication. DNA was precipitated from the lysates using 0.15% polyethyleneimine and lysates were clarified by centrifugation at ∼50 k(×***g***). The hexahistidine-tagged proteins were immobilized on nickel–sepharose and eluted using a binding buffer with increasing amounts of imidazole to 250 mM. The eluted proteins were cleaved with TEV protease at 4°C overnight and further purified by SEC using an S200 HiLoad 16/60 Superdex column equilibrated in buffer containing 50 mM HEPES pH 7.5, 300 mM NaCl and 0.5 mM TCEP. A final clean-up step was performed by passing elution fractions containing the protein of interest over a column of nickel–sepharose and collecting the flow-through as needed. Proteins were concentrated using centrifugal ultrafiltration with a neutral mixture of 1 : 1 l-arginine/l-glutamate and/or dithiothreitol added as needed to maintain solubility (details of final buffers used in crystallization trials for each protein are detailed in Supplementary Table S4). Protein concentration was determined by UV absorbance at 280 nm. Purity of >95% was verified by SDS–PAGE and construct identities were verified by MS. Protein was either used fresh or flash frozen in liquid nitrogen and stored at −80°C.

### Crystallization and diffraction data collection

Crystals were grown using the sitting-drop vapour-diffusion method (full conditions for each protein are listed in Supplementary Table S4) and cryoprotected in mother liquor plus 25% ethylene glycol before vitrification in liquid nitrogen. Typically, four crystallization screens were attempted for each protein (JCSG7, HCS3, HIN3 and LFS6 produced by Molecular Dimensions, Suffolk U.K.). 150 nl drops at three ratios of mother liquor to protein solution, 2 : 1, 1 : 1 and 1 : 2 were equilibrated against mother liquor at 20 and 4°C. For KCTD10, crystals were briefly soaked in mother liquor supplemented with 5 mM thiomersal prior to cryoprotection. Diffraction data were collected at the Diamond Light Source beamlines I03 and I04 using synchrotron radiation at 100 K.

### Structure determination

Data were integrated using XDS [37] and scaled with AIMLESS [38] as part of the CCP4 software suite [39]. Initial phases were either estimated by molecular replacement using PHASER [40] or experimentally determined from anomalous data using PHENIX.AUTOSOL [37]. Automated model building was performed using PHENIX.AUTOBUILD [41]. Alternatively, MR-Rosetta [42] was used for integrated structure modelling, molecular replacement, model building, density modification and refinement. Manual model building was performed in Coot [43] and refinement completed using PHENIX.REFINE employing NCS restraints as appropriate [41]. MOLPROBITY [44] was used to validate models during building and refinement.

### Isothermal titration calorimetry

Proteins were dialyzed overnight into ITC buffer (50 mM HEPES pH 7.5, 150 mM NaCl, 1 mM TCEP and 5% glycerol) using D-Tube^™^ Midi Dialyzers with 3.5 kDa molecular mass cut-off (Merck). ITC experiments were performed using a Microcal VP-ITC microcalorimeter. All KCTD family proteins were titrated into Cul3, except for KCTD17 which produced better data when loaded into the cell and titrated with Cul3 from the syringe. Experiments were conducted at 15°C except for KCTD5 which was conducted at 25°C. Experimental data were fitted to the single-binding site model implemented in the Origin software package provided with the instrument.

### SEC–MALS

SEC performed with a Shodex KW-803 column on a Dionex micro-HPLC system was coupled to the analytics of a Tetra detector (Malvern Instruments Ltd.), which monitors the refractive index, low (7°) and right angle light scattering, viscosity and UV absorbance. Experiments were performed in 50 mM HEPES pH 7.5, 150 mM NaCl, 1 mM TCEP unless indicated otherwise at a flow rate of 0.5 ml/min. All proteins were injected at a final concentration of 50 µM. For complexes, Cul3 and KCTD proteins were mixed at a molar ratio of 1 : 1.

### Native mass spectrometry

Proteins were desalted and exchanged into volatile buffer as follows: 75 µl of KCTD17 at 2 mg/ml was applied to Micro BioSpin 6 size-exclusion columns (Bio-Rad) previously equilibrated with 50 mM ammonium acetate pH 6.5 buffer. Samples were exchanged into this buffer following the manufacturer's instructions. The eluent was collected and subjected to two further rounds of size exclusion and held on ice prior to MS analysis. MS was performed using an Agilent 6530 QTOF mass spectrometer with the following acquisition parameters: ion mode positive; detector mode 1 GHz; scan range 100 *m/z* to 20 000 *m/z*; collision cell off; capillary 3500 V; fragmentor 430 V; skimmer 65 V; octopole rf 750 V; drying gas 325°C; drying gas 5 L/min; nebulizer 17 psi. The instrument was configured with a standard ESI source and a 30 gauge stainless steel nebulizer needle. Sample for analysis was transferred to a 200 µl gas tight syringe (Hamilton, Switzerland) and delivered into the mass spectrometer by direct infusion at 6 µl/min. Charge assignment was made using a charge table with 60 times the observed monomer mass for KCTD17 as the input. The charge radius of the complex was calculated using the method of Testa et al. [32] and the most abundant charge state (+79).

### SAXS

BTB domain proteins were dialyzed overnight at 4°C into 50 mM HEPES pH, 150 mM NaCl, 0.5 mM TCEP. A sample of the dialysis buffer was used for buffer subtraction during data reduction. For complexes, BTBs and Cul3 were co-purified over an S200 gel filtration column. An additional ∼10% of Cul3 was added to slightly oversaturate hetero-multimers and then 30 µl of 10 mg/ml complex was injected onto a Shodex KW404 size-exclusion column at 20°C using 50 mM HEPES pH 7.5, 150 mM NaCl, 1 mM TCEP, 5% glycerol as the running buffer. Data were collected on freshly isolated complex peaks and size-exclusion running buffer was used for buffer subtraction. The leading edge of the gel filtration peak was used to analyse of the largest complexes. Small-angle X-ray scattering data were collected at the B21 bending magnet instrument at Diamond Light Source (Harwell, U.K.). The output flow from the Agilent HPLC column was directed through a 1.6 mm diameter quartz capillary cell which was held in vacuum. The flow rate was set to 0.16 ml/min. The cell was illuminated with an X-ray beam of 1 Å wavelength. X-ray data were collected from the size-exclusion peaks and the running buffer before and after the peak. A Pilatus 2M two-dimensional detector was used to collect 10 frame exposures of 10 s from each sample and the corresponding buffer. The detector was placed at 3.9 m from the sample, giving a useful q-range of 0.005 Å^−1^ < 0.4 Å^−1^, where *q* = 4π sin θ/λ, where 2θ is the scattering angle and λ is the wavelength. Two-dimensional data reduction consisted of normalization for beam current and sample transmission, radial sector integration, background buffer subtraction and averaging. Further data analyses, such as scaling, merging and Guinier analysis were performed in Scatter (DLS, U.K.). Experimental curves were compared with generated models and crystallographic structures using CRYSOL [45]. Data for the 6His-KCTD17 60-mer were analysed using Igor Pro (Wavemetrics, OH) software, with data-fitting conducted in the NIST Igor macro routines [46]. A core-shell model was used for this system, with the core scattering length density set to be equal to that of the solvent (i.e. reproducing the scattering from a hollow sphere).

## Abbreviations

BTB: broad-complex, Tramtrack, and Bric à brac
CRL: Cullin-RING ligase
CTD: C-terminal domain
GABA: gamma-aminobutyric acid
ITC: isothermal titration calorimetry
LB: Luria-Bertani broth
MS: mass spectrometry
KCTD: potassium (K+) Channel Tetramerization Domain
SAXS: small angle X-ray scattering
SEC–MALS: size-exclusion chromatography combined with multi-angle light scattering
TCEP: tris(2-carboxyethyl)phosphine
TEV: Tobacco Etch Virus
