# Supplementary material for: Structural complexity in the KCTD family of Cullin3-dependent E3 ubiquitin ligases
Source: Biochem J. 2017 Nov 1;474(22):3747–61. doi: 10.1042/BCJ20170527 (PMC5664961; doi:10.1042/BCJ20170527)
Supplement: Supplementary Tables and Figures [file BCJ-474-3747-s1.pdf]

Structural Complexity in the KCTD Family of Cullin3-Dependent E3 Ubiquitin Ligases

Daniel M. Pinkas[1], Caroline E. Sanvitale[1], Joshua C. Bufton[1], Fiona J. Sorrell[1], Nicolae Solcan[1,2], Rod Chalk[1], James Doutch[3], Alex N. Bullock[1]*

SUPPLEMENTARY MATERIAL

SUPPLEMENTARY TABLES

Table S1. Crystallographic data collection and refinement statistics

	SHKBP1	KCTD10	KCTD13	KCTD16	KCTD17
PDB Code	4CRH	5FTA	4UIJ	5A15	5A6R
Beamline[a]	I04	I04	I03	I03	I03
Wavelength (Å)	0.976	0.976	0.976	0.976	0.976
Resolution range (Å) [b]	35.65 - 1.72<br>(1.78 - 1.72)	17.77 - 2.64<br>(2.73 - 2.64)	37.42 - 2.70<br>(2.80 - 2.70)	34.65 - 2.76<br>(2.86 - 2.76)	86.16 - 2.85<br>(2.96 - 2.85)
Space group	$P6_5$	$P2_1$	$P2_1$	$P1$	$F432$
Unit cell *a, b ,c* (Å)<br>α, β, γ (°)	71.29, 71.29, 38.41<br>90, 90, 120	48.15, 83.66, 58.68<br>90, 95.18, 90	49.45, 58.83, 87.17<br>90, 105.41, 90	57.79, 69.37, 134.24<br>102.45, 95.72, 90.71	285.743, 285.743, 285.743<br>90, 90, 90
Total reflections[b]	66691 (6723)	91190 (7864)	66952 (6535)	177717 (18250)	329351 (33863)
Unique reflections[b]	11953 (1191)	13616 (1347)	13421 (1314)	50927 (5093)	23906 (2340)
Multiplicity[b]	5.6 (5.6)	6.7 (5.8)	5.0 (5.0)	3.5 (3.6)	13.8 (14.5)
Completeness (%) [b]	99.78 (99.67)	99.56 (99.19)	99.86 (99.70)	97.93 (98.30)	99.91 (100.00)
$\langle I \rangle / \sigma(I)$[b]	21.74 (2.50)	16.13 (2.58)	8.25 (1.87)	13.74 (2.23)	17.50 (1.90)
Wilson B-factor	23.58	47.62	57.2	82.05	97.29
R_{merge}[b]	0.049 (0.674)	0.098 (0.614)	0.119 (0.843)	0.047 (0.601)	0.095 (1.80)
R_{meas}	0.054	0.106	0.133	0.055	0.099
$CC_{1/2}$[b]	0.999 (0.784)	0.998 (0.829)	0.993 (0.717)	0.999 (0.854)	0.996 (0.635)
CC*[b]	1.000 (0.938)	0.999 (0.952)	0.998 (0.914)	1.000 (0.960)	0.999 (0.881)
Nr. of reflections for R_{free}[b]	572 (130)	681 (138)	714 (158)	2456 (129)	1211 (129)
R_{work}[b]	0.164 (0.239)	0.226 (0.290)	0.235 (0.322)	0.217 (0.336)	0.220 (0.340)
R_{free}[b]	0.193 (0.244)	0.258 (0.317)	0.279 (0.353)	0.259 (0.383)	0.242 (0.370)
Nr. of non-hydrogen atoms	870	3104	3190	11810	4062
macromolecules	771	3068	3152	11810	4054
ligands	0	4	4	0	0
water	98	32	34	0	8
Protein residues	94	398	410	1425	507
R.M.S. (bonds)	0.008	0.003	0.003	0.003	0.003
R.M.S. (angles)	1.21	0.85	0.62	0.79	0.55
Ramachandran favored (%)	100	99	98	98	98
Ramachandran allowed (%)	0	1	2	2	2
Ramachandran outliers (%)	0	0	0	0	0
Clashscore	1.29	0.98	0.16	1.28	1.00
Average B-factor (Å^2)	27.7	49.9	61.3	106.3	99.5
macromolecules	26.5	49.9	61.4	106.3	99.5
ligands	-	63.9	78.9	-	-
solvent	37.4	42.8	52.4	-	71.1

[a]Diamond Light Source, Harwell UK
[b]Values in parentheses indicate data for the highest resolution shell

Table S2. SAXS data collection and calculated parameters

(A)

Data collection parameters	
Instrument	Bending magnet B21, Diamond Light Source, Harwell (UK)
Energy	12.4keV from double crystal monochromator
Beam geometry	Focussed on detector ~0.2mm x 0.2mm, at sample position ~ 6mm x 1mm
Q range ($Å^{-1}$)	~0.005 – 0.4
Temperature (K)	293
Exposure times	Frames 10s
Software Employed	
Primary data reduction	DAWN pipeline (Diamond Light Source, UK)
Data processing	Scatter v3.0 (Diamond Light Source, UK)
Computation of model intensities	CRYSOL
Form factor modelling	NIST macro suite for Igor (Wavemetrics, OH)

(B)

	$KCTD16^{BTB}$	$KCTD10^{BTB}$	$KCTD17^{BTB}$	$SHKBP1^{BTB}$ + Cul3	$KCTD13^{BTB}$ + Cul3	$KCTD17^{BTB}$ +Cul3
*Structural parameters**						
Rg (Å) (from Guinier)	29.9	24.4	65.6	67.5	61.2	59.4
Porod volume estimate ($Å^3$)	147200	84300	482600	419000	327200	1290200
Molecular weight estimate (kDa)	86.6	49.6	283.9	246.5	192.5	759

* Data not collected on absolute scale; intensity in arbitrary units. Weak scattering in the high q region may affect the estimates of Porod volume and molecular weight.

Table S3. ITC binding data

Peptide	K_D (nM)	$K_B \times 10^7$ (M^{-1})	ΔH^{obs} (kcal/mol)	$T\Delta S$ (kcal/mol)	ΔG (kcal/mol)	N *
$SHKBP1^{BTB}$	87	1.15 ± 0.25	-4.3 ± 0.1	4.98	-9.30	0.92
$KCTD10^{BTB}$	460	0.22 ± 0.04	-10.7 ± 0.3	-2.30	-8.63	0.62
$KCTD13^{BTB}$	100	1.01 ± 0.22	-8.6 ± 0.2	0.67	-9.24	0.87
KCTD16		No binding				
$KCTD17^{BTB}$	7	14.1 ± 9.9	-7.2 ± 0.1	3.51	-10.7	
$KCTD17^{BTB\text{-}CTD}$	12	8.66 ± 2.9	-3.1 ± 0.1	7.34	-10.5	2.3
$KCTD5^{BTB}$	55	1.80 ± 0.21	-17.2 ± 0.2	-7.27	-9.91	0.81
$KCTD5^{BTB\text{-}CTD}$	1	79.5 ± 62.9	-16.9 ± 0.3	-4.80	-12.1	0.94

*Stoichiometry determined from a single binding site model. ITC binding curves for these data are shown in Figure 5.

Table S4. Crystallization conditions

	KCTD10	KCTD13	KCTD16	KCTD17	SHKBP1
Protein Buffer	50 mM HEPES pH 7.5, 300 mM NaCl and 5% glycerol	50 mM HEPES pH 7.5, 300 mM NaCl and 5% glycerol	50 mM HEPES pH 7.5, 300 mM NaCl, 0.5 mM TCEP and 5% glycerol	50 mM HEPES pH 7.5, 300 mM NaCl and 5% glycerol	50 mM HEPES pH 7.5, 400 mM NaCl, 2.5% glycerol, 0.5 mM TCEP, 5 mM DTT, 20 mM L-Arginine and 20 mM L-Glutamate
Protein Concentration (mg/mL)	17	2.75	13	30	7.7
Mother Liquor	20% PEG3350, 0.1M tris pH 8.4, 0.2 M magnesium chloride	1.6 M magnesium sulfate, 0.1 M MES pH 6.5	21% PEG3350, 0.1 M tris pH 5.5	18% MPD, 0.1 tris pH 8.5	17% PEG MME 2000, 0.1 M tris pH 8.4, 0.15 M Trimethlyalmine N-oxide
Drop Volume (nL)	150	150	300	150	150
Protein:Mother Liquor Ratio	1:2	1:1	2:1	2:1	2:1
Crystallization Temperature (°C)	4	20	20	4	4

SUPPLEMENTARY FIGURES

Figure S1.

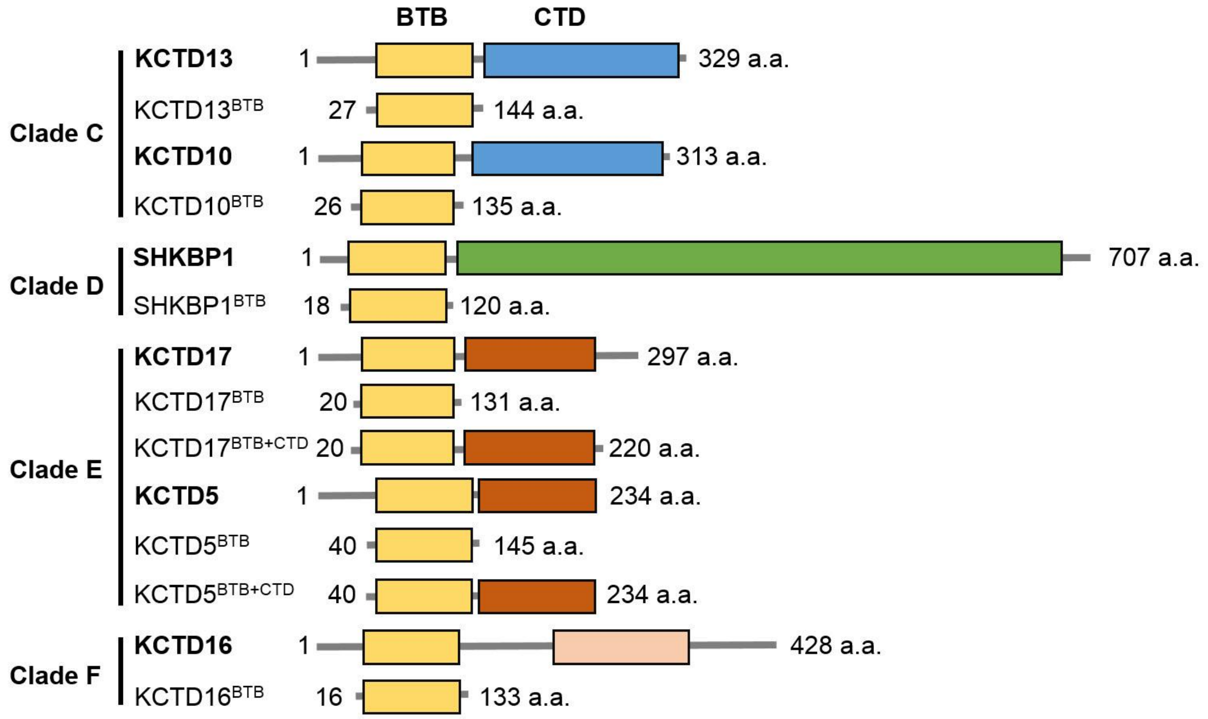

Figure S1. KCTD family protein domain organization and construct boundaries. Details are provided for selected KCTD family proteins used in this study. Native full length proteins are indicated in bold; expression constructs are shown below.

Figure S1. Guinier Plots

KCTD10^{BTB}

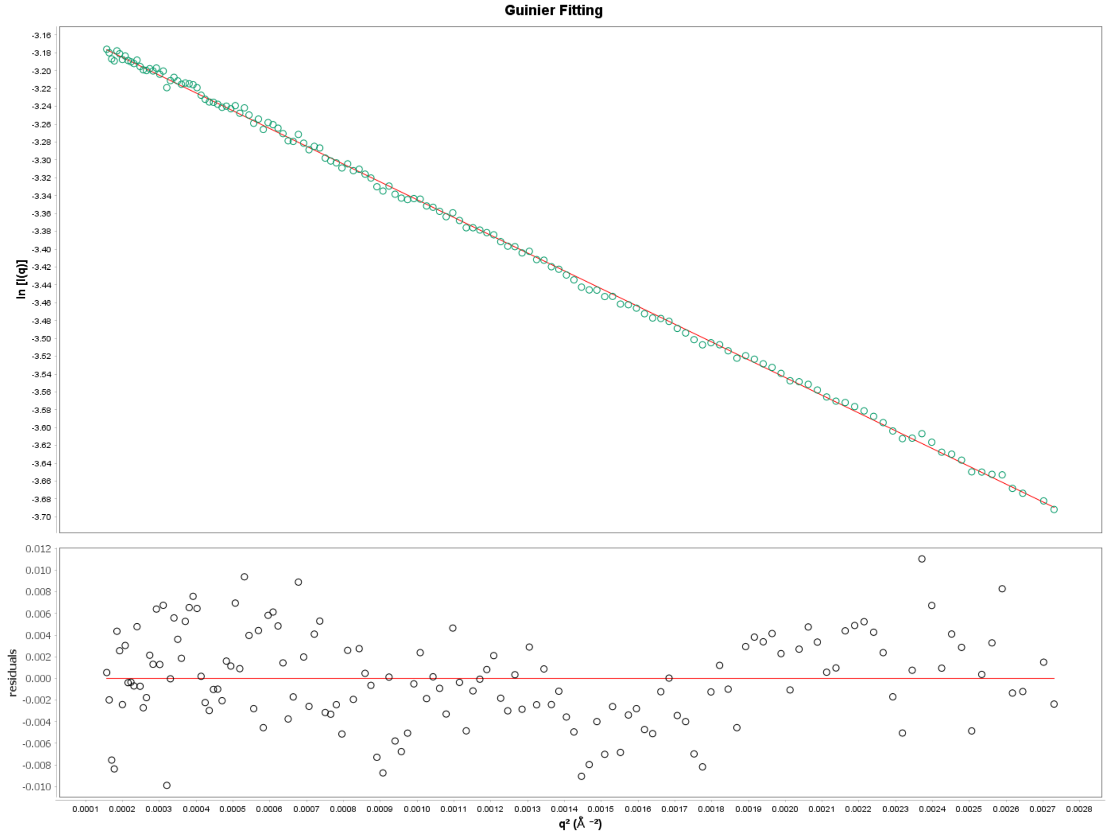

KCTD16^{BTB}

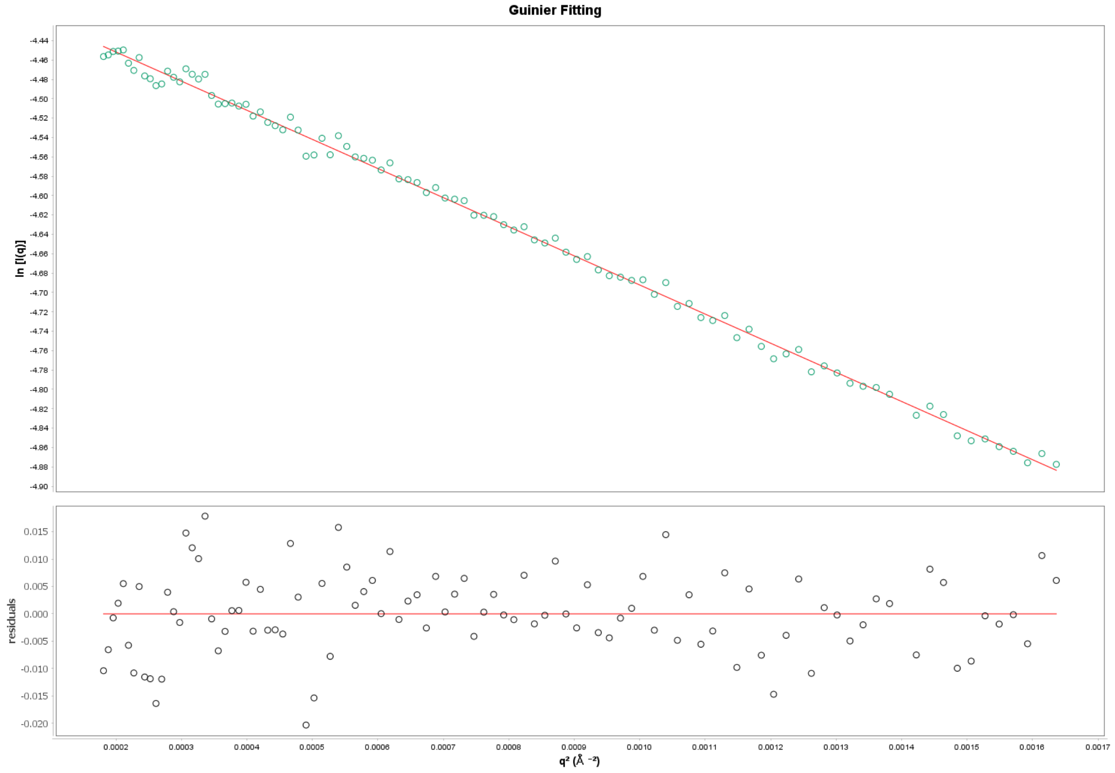

KCTD17^{BTB}

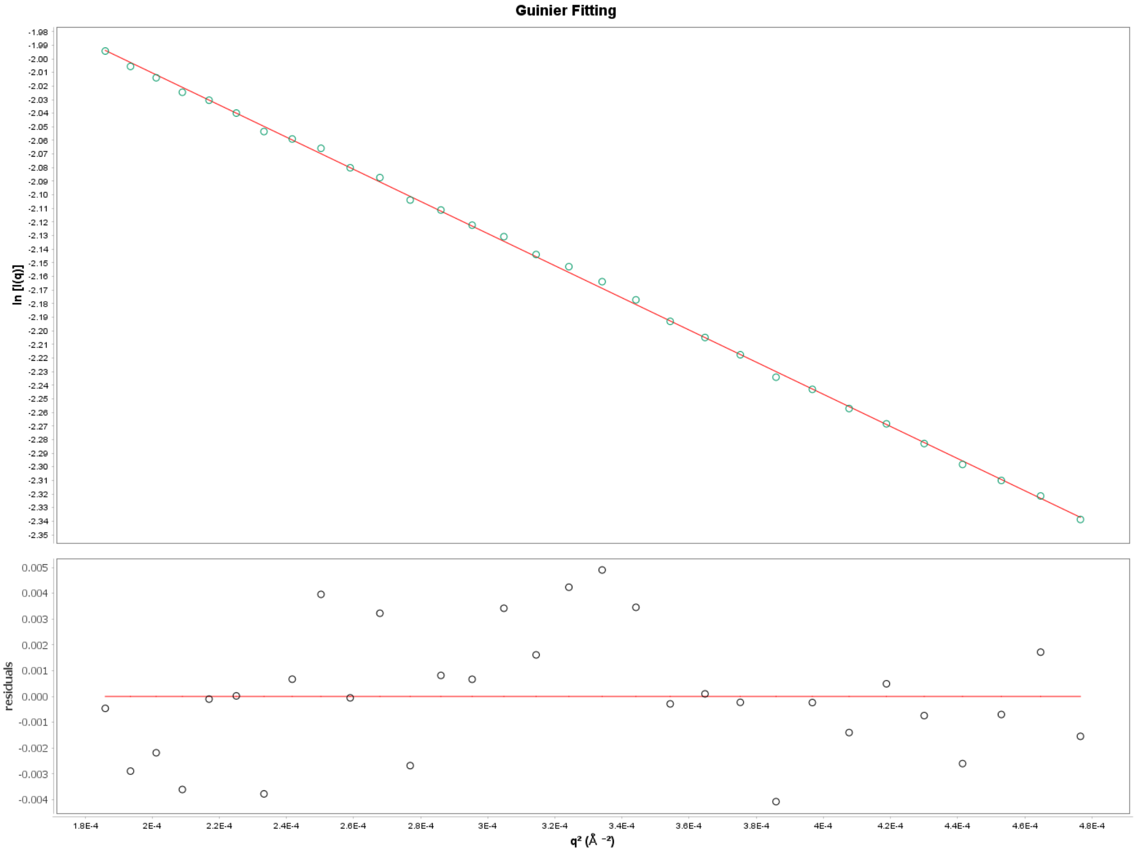

KCTD17$^{BTB+CTD}$ in complex with Cul3

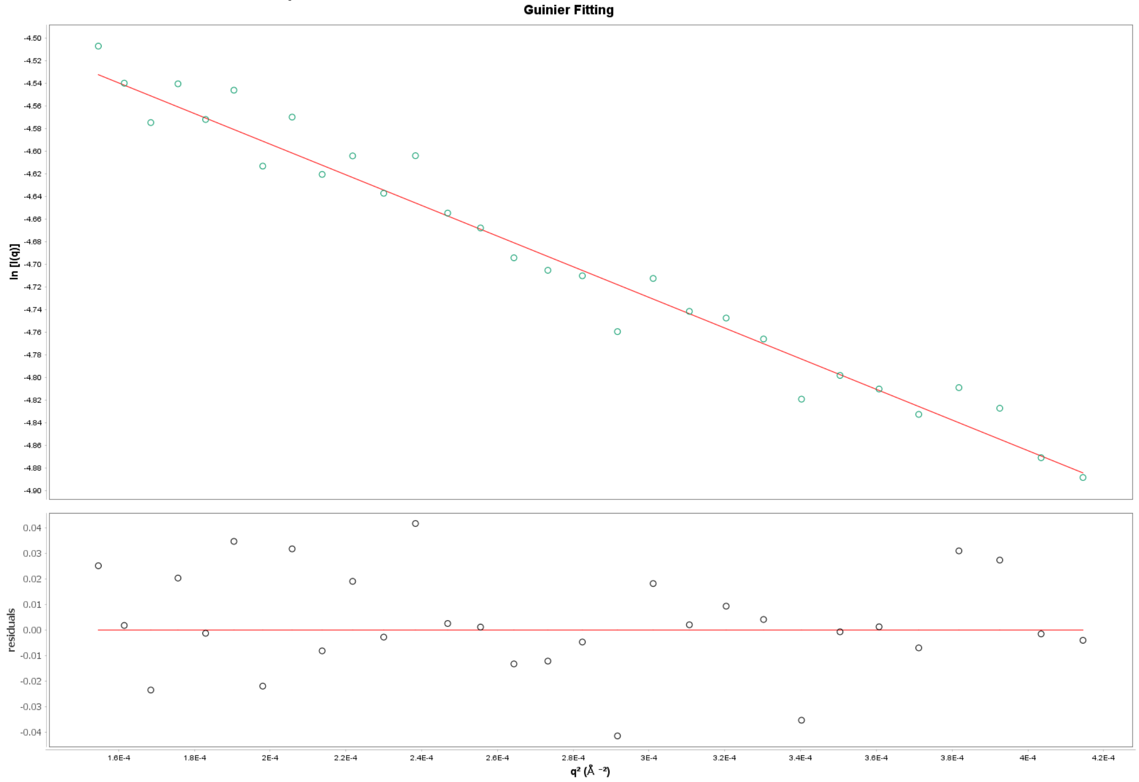

SHKBP1^{BTB} in complex with Cul3

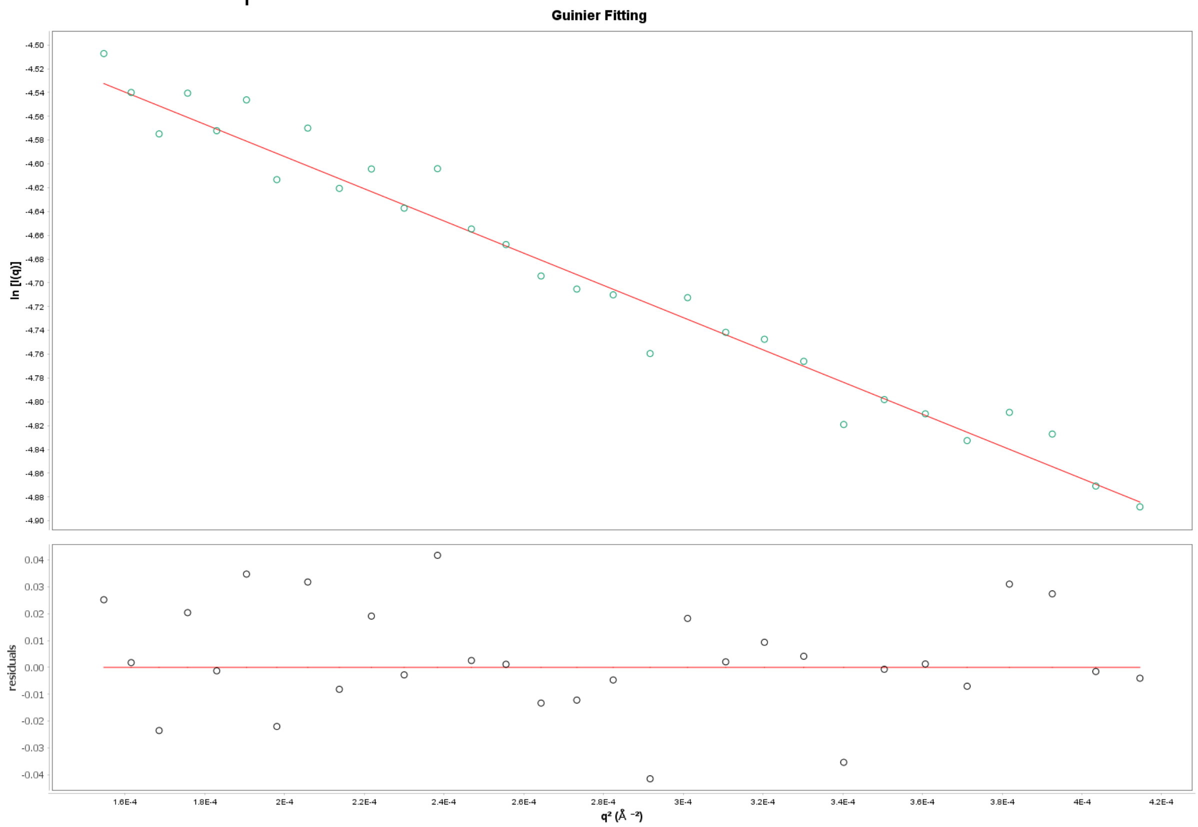

KCTD13^{BTB} in complex with Cul3

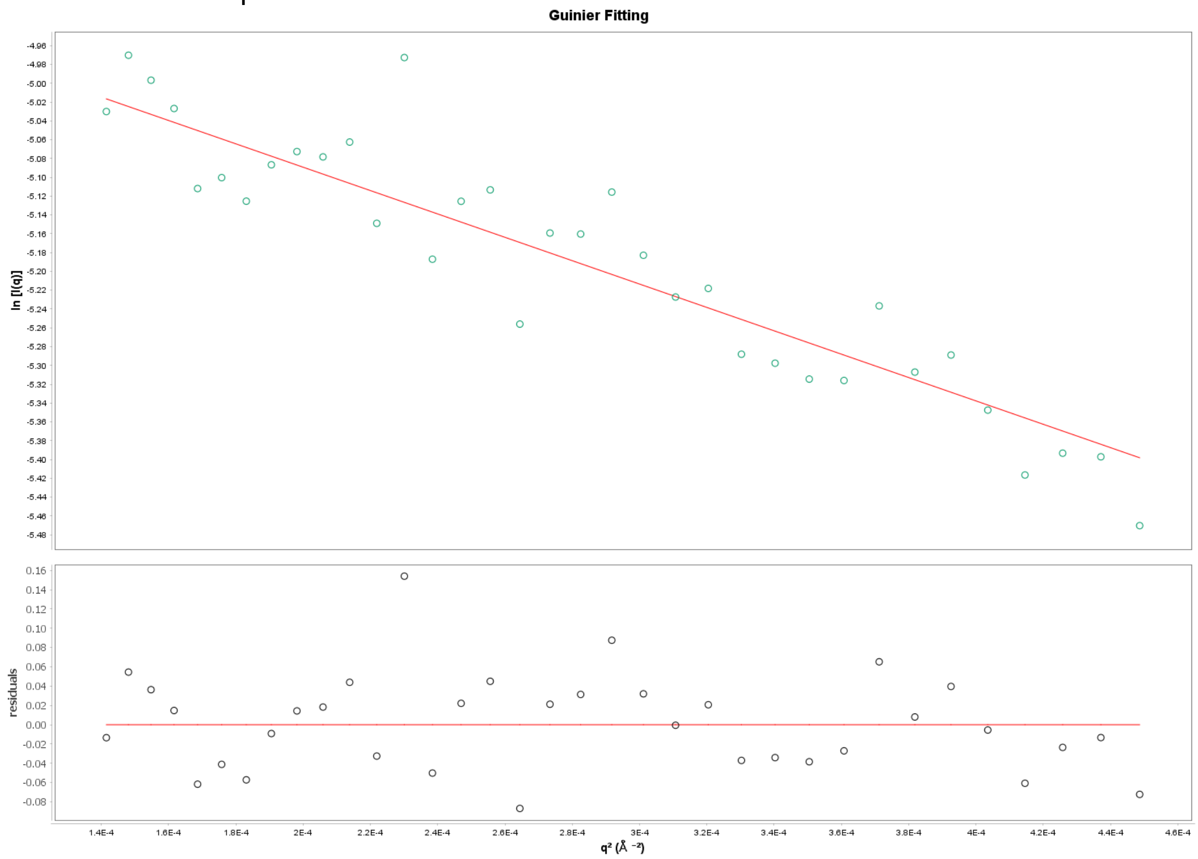

Figure S3.

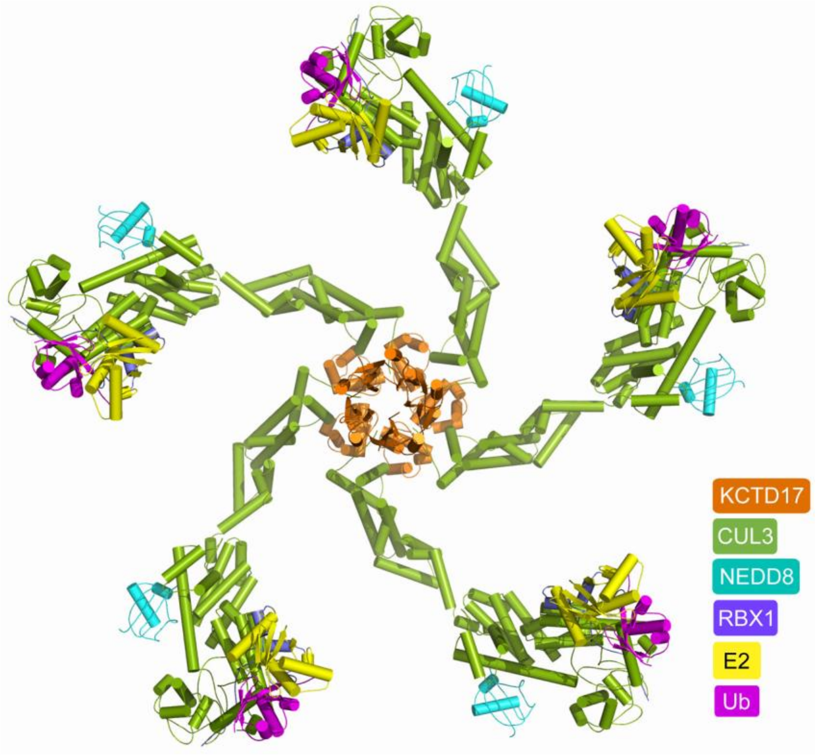

Figure S3. Model of the KCTD17 ubiquitin ligase complex with charged E2-ubiquitin pairs.
The model shown in Fig. 7C is redrawn as viewed from the top of the CTD domains of KCTD17.
